# Supplementary material for: Comparing level of food insecurity between households with and without home gardening practices in Zege, Amhara region, North West Ethiopia: Community based study
Source: PLoS One. 2022 Dec 21;17(12):e0279392. doi: 10.1371/journal.pone.0279392 (PMC9770380; doi:10.1371/journal.pone.0279392)
Supplement: Attachment — Submitted filename: Point-by-point response.docx [file pone.0279392.s002.docx]

Point-by-point response to reviewers’ comments

Manuscript ID: **PONE-D-22-10969**

Manuscript Title: **Comparing level of food insecurity between households with and without home gardening practices in Zege, Amhara region, North West Ethiopia: Community based study**

Dear Editor and the reviewers,

I would like to express my acknowledgements to you for your contribution given us by reviewing the manuscript. It is quite clear that the quality and readability of the manuscript will be increased once we addressed the comments and suggestion given by all of you. All the give comments and suggestions are addressed and the required responses and amendments are given in the following table.

**Editor comments**

| **Reviewer/Editor Comments** | **Authors response** | **Page number** |
| --- | --- | --- |
| Please ensure that your manuscript meets PLOS ONE's style requirements, including those for file naming. | Addressed as per PLOS guideline |  |
| We noticed you have some minor occurrence of overlapping text with the following previous publication(s), which needs to be addressed: (***published reticles websites are given***). | **Addressed by rephrase those identified overlapping as follows:**  **Previous:** Evidence from Ethiopia showed that households with large family size, who cannot read and write and old household heads are more likely to be food insecure than their counter parties. Same study showed small land size, lack of livestock, not having confidence to overcome food insecurity of HHs, borrowing money from informal rural money lenders and not using farm input by the HHs are significantly associated with food insecurity  **Revised:** Studies from Ethiopia showed that there are different factors that affect household food security. These factors include; having large family size, those who cannot read and write, and households lead by old household heads. In addition, having small size farm lands, lack of livestock, those who do not have confidence to overcome food insecurity of the households, not using farm inputs by the households are other factors affecting food insecurity.  **Previous**: In pooled regression analyses from eight different countries in different parts of the world showed that a 10-point increase in food access insecurity score was significantly associated with a 0.20 SD decrease in height-for-age Z score (stunting).  **Revised:** According to the study results in rural, urban, and peri-urban areas of eight countries of the world, when there is an increase in food access insecurity score, child height-for-age z-score or standard deviation is significantly decrease or when there is an increase in food access insecurity score, the child become more stunted.  **Previous:** Another study done in Kenya showed that the risk of stunting increased by 12% among children from food insecure households. When the joint effect of food security and wealth status was assessed, the risk of stunting increased significantly by 19 and 22% among children from moderately food insecure and severely food insecure households  **Revised:** Study from Kenya showed that children from food insecure households are more at risk of becoming stunted to 12 percent than their counter parts. With this same study, the risk of stunting increased by 19% from moderately food insecure households and by 22% from severely food insecure households when the effects of food security and wealth status together assessed.  **Previous:** It is already understood that agricultural growth itself does not necessarily lead to improved nutrition and governments, donors, UN agencies and NGOs have been promoting home gardens to achieve better family nutrition for many years, mostly with positive results.  **Revised:** There is an agreement so far where agricultural growth by itself does not necessarily bring about family nutrition improvements. Home gardening practice is believed to have great role in family nutrition improvements and has positive results. Hence, responsible agents like governments, donors, UN agencies and NGOs need to invest to promoting it.  **Previous:** Study result showed a positive significant relationship between home gardening and household food security status. This same study established that home gardening does not only contribute to their house hold food supply but also their income  **Revised:** Evidence reveled that home gardening practice positively affect households living standard by improving household food security status and household income. | Page 4, 5 |
| We note that you have indicated that data from this study are available upon request. PLOS only allows data to be available upon request if there are legal or ethical restrictions on sharing data publicly. | The authors will give data to PLOS ONE and archive it for free access. |  |
| In your Data Availability statement, you have not specified where the minimal data set underlying the results described in your manuscript can be found. | I upload our study’s minimal underlying data set as Supporting Information files |  |
| Please ensure that you refer to Table 3 in your text as, if accepted, production will need this reference to link the reader to the Table | **Accepted:** I referred Table 3 In the text on page 15 |  |

**Reviewer 1 comments**

| **Reviewer/Editor Comments** | **Authors response** | **Page number** |
| --- | --- | --- |
| I would like to know how the dependent variable (food security/ insecurity) has been identified? | **Addressed and revised in the following way:**  Household food-security (access) information was col­lected by using the questionnaire adopted from the House­hold Food Insecurity Access Scale, which was developed by the Food and Nutrition Technical Assistance project. This instrument consists of nine questions that measure uncertainty on obtaining food, limited access to high-quality foods, and reduction in food quantity in the past 4 weeks. The precoded options were never (0 points), rarely (once or twice in the past 4 weeks; 1 point), sometimes (three to ten times in the past 4 weeks; 2 points), and often (more than ten times in the past 4 weeks; 3 points). Scores for answers to these ques­tions were summed (0–27), and thus a household experiences none of the food insecurity conditions, or just experiences worry, but rarely categorized as food secure otherwise food insecure household. |  |
| In literature there are several indicators for monitoring household poverty levels, food poverty and access to food. Authors should better explain this point in a section devoted to the literature review. | **The following points are included in the introduction part of the manuscript:**  Because it is a complex and multidimensional concept, measuring food insecurity has been an ongoing challenge to researchers and practitioners alike. Despite apparent empirical strength, the operationalization of the food security concept still presents many challenges. Having similar definitions for food security, measurements and assessments methods is difficult and can differ considerably, even within the boundaries of the qualitative and quantitative traditions. The available food security measurement methods considered per capita calorie, number of foods or food groups, proportion of income people spend on food, behavior of people related to food consumption and behavioral and psychological measures in relation with food consumption are the main concepts for measurement. Household Food Insecurity Access Scale (HFIAS), food consumption score, household expenditure survey, calorie intake/food energy availability per capita, and coping strategy are some to mention from the different methods used by different experts to assess food security/insecurity level. |  |
| Moreover, the aim of the paper and the strategy followed in the paper should be better identified in the introduction section. | **Objective:** This research tried to compare proportion of food insecurity between households who practicing and non-practicing of home gardening and the associated factors of food insecurity among these households. |  |
| Finally, I suggest authors to provide a proofread of the paper since there are some typos (eg. In line 44 “developing counties” should be “developing countries”. | Revision has been done to address your comments. |  |

Reviewer 2 comments

| **Reviewer/Editor Comments** | **Authors response** | **Page number** |
| --- | --- | --- |
| The authors have to precisely define the food security/insecurity variable. | ***Addressed and revised in the following way:***  Household food-security (access) information was col­lected by using the questionnaire adopted from the House­hold Food Insecurity Access Scale, which was developed by the Food and Nutrition Technical Assistance project. This instrument consists of nine questions that measure uncertainty on obtaining food, limited access to high-quality foods, and reduction in food quantity in the past 4 weeks. The precoded options were never (0 points), rarely (once or twice in the past 4 weeks; 1 point), sometimes (three to ten times in the past 4 weeks; 2 points), and often (more than ten times in the past 4 weeks; 3 points). Scores for answers to these ques­tions were summed (0–27), and thus a household experiences none of the food insecurity conditions, or just experiences worry, but rarely categorized as food secure otherwise food insecure household. |  |
| The introduction section has to be revised, there is need to describe clearly the aim of the paper and the methods and data used to monitor poverty levels, food poverty and access to food. | **I revised the objective as follow:** This research tried to compare proportion of food insecurity between households who practicing and non-practicing of home gardening and the associated factors of food insecurity among these households.  However, the description of methods and data used to monitor poverty levels, food poverty and access to food is addressed in the method section of the manuscript.  **The following points also included to address the introduction section of your comments:**  Because it is a complex and multidimensional concept, measuring food insecurity has been an ongoing challenge to researchers and practitioners alike. Despite apparent empirical strength, the operationalization of the food security concept still presents many challenges. Having similar definitions for food security, measurements and assessments methods is difficult and can differ considerably, even within the boundaries of the qualitative and quantitative traditions. The available food security measurement methods considered per capita calorie, number of foods or food groups, proportion of income people spend on food, behavior of people related to food consumption and behavioral and psychological measures in relation with food consumption are the main concepts for measurement. Household Food Insecurity Access Scale (HFIAS), food consumption score, household expenditure survey, calorie intake/food energy availability per capita, and coping strategy are some to mention from the different methods used by different experts to assess food security/insecurity level. |  |
